# Efficient delivery of the immunodominant genes of African swine fever virus by adeno-associated virus serotype 2

**DOI:** 10.14202/vetworld.2023.2425-2430

**Published:** 2023-12-06

**Authors:** Rustam Ravilov, Antonina Galeeva, Gennadiy Frolov, Marina Efimova, Elena Zakirova, Albert Rizvanov, Almaz Hisamutdinov, Lenar Garipov, Danil Mingaleev

**Affiliations:** 1 Kazan State Academy of Veterinary Medicine named after N.E. Bauman, Kazan, Russia; 2 Federal Center for Toxicological, Radiation and Biological Safety, Kazan, Russia; 3 Laboratory of Regenerative Veterinary Medicine, Kazan (Volga region) Federal University, Institute of Fundamental Medicine and Biology, Kazan, Russia; 4 Main Directorate of Veterinary Medicine, Ministry of the Tatarstan Republic, Kazan, Russia; 5 Ministry of Agriculture and Food of Tatarstan Republic, Kazan, Russia.

**Keywords:** adeno-associated virus, African swine fever virus, gene delivery, virally vectored vaccine

## Abstract

**Background and Aim:**

Adeno-associated virus serotype 2 (AAV2) represents a promising basis for developing a virus-vector vaccine against African swine fever (ASF). This study aimed to create genetic constructs based on AAV2 to deliver the immunodominant genes of ASF virus (ASFV) and to evaluate their functionality *in vitro*. The efficiency and specificity of transgene expression, as well as their non-toxicity in cells of target animals, were evaluated.

**Materials and Methods:**

Bioinformatics analysis methods were used to identify the immunodominant genes of ASFV. The target genes *B646L*, *E183L*, *CP204L*, and *CP530R* were identified and subsequently cloned into the pAAV-MCS vector. Assembly of recombinant AAV2 (rAAV2) was performed by cotransfection of AAV293 cells with the following plasmids: pAAV-MCS with the gene of interest, envelope, and packaging. Quantitative polymerase chain reaction was used to determine the AAV2 titer. The functionality of the constructs was evaluated in HEK293 and SPEV cells by determining the presence of mature proteins in the cell lysate and the expression levels of messenger RNA. The specificity of the target proteins in cell lysates was confirmed by Western blotting.

**Results:**

The proposed AAV2 assembly protocol makes it possible to achieve a concentration of mature viral particles of at least 280 billion/mL of virus-containing material. The rAAV2 could effectively transduce host SPEV cells. The expression of both cistrons was detectable during the transduction of cells; therefore, the combined expression of immunogens in the cells of target animals should be possible using this method.

**Conclusion:**

This study demonstrated the potential of using genetic constructs based on AAV2 for the delivery of ASFV genes *in vitro*.

## Introduction

One of the most acute problems in world of agriculture is the development of an effective vaccine against African swine fever (ASF), which is caused by ASF virus (ASFV). Traditional methods do not allow the development of vaccines that provide a wide range of cross-immune responses to antigenically changing strains of a pathogen. The development of a vaccine against ASF is largely hampered by large knowledge gaps in the functions of the viral genes and the roles of its proteins and their combinations in virulence, immunopathology, and protection [1–3].

African swine fever virus, the only member of the *Asfarviridae* family, is a complex, enveloped, and double-stranded DNA virus [4, 5]. The length of the viral genome varies from 170 kb to 194 kb, depending on the virus strain, and encodes at least 168 viral proteins, including 68 structural and 100 non-structural proteins [6, 7]. The functions of most ASFV proteins are unclear, although it is the subject of many ongoing studies [8]. To date, many potentially protective proteins encoded by the ASFV genes have been screened. The recombinant p30, p72, p54, and p22 proteins (encoded by the *CP204L*, *B646L*, *E183L*, and *KP177R* genes, respectively) have been demonstrated to induce the formation of neutralizing antibodies in pigs, but did not protect the animals from lethal infection [9]. In contrast, p54 and p30 fusion proteins also induced neutralizing antibodies in pigs, but effectively protected the animals from severe disease after infection [10]. CD2v (encoded by *EP402R*) induces antibodies in pigs that inhibit hemadsorption of erythrocytes to the surface of ASFV-infected cells and provides partial protection against virulent ASFV [2]. DNA vaccines have shown high efficiency: immunization with a collection of ASFV genomic DNA fragments stimulated the appearance of immunity in 60% of pigs in a previous study by Sunwoo *et al*. [11].

The potential of viral vectors as carriers for delivering desired immunogens has been demonstrated in previous studies [3, 12–14]. To date, adeno-associated virus (AAV) is the most extensively studied virus and has been included in more than 100 clinical trials of gene therapy and prevention due to its broad tropism for many cell types, stable gene expression, and safety [15, 16]. However, the disadvantages of all viral vectors, including AAV, include host immune responses to a capsid or transgene, appropriate transduction of the target tissue, capacity limitations, and the difficulty of obtaining vectors with high titers economically in a short-time frame [17]. In recent years, to solve some of the identified problems and obtain maximum effects from combined gene therapy or prevention, it has become necessary to create more complex polycistronic viral vectors. In particular, to induce an immune response against infectious agents of a complex antigenic structure carrying several epitopes, it is necessary to deliver more than one gene to the host cells. The simultaneous coexpression of several viral antigens can provide a more intense immune response, allowing a reduction in the vector dose without any loss of efficiency [18]. However, clinical trials to evaluate the efficacy and safety of two or more separate AAV monocistronic constructs are significantly more time-and cost-intensive than those for single polycistronic constructs.

In this study, we aimed to create genetic constructs based on AAV serotype 2 to deliver the immunodominant genes of ASFV and to evaluate their efficiency *in vitro*. The results of this study will be helpful in the assessment of the suitability of viral vector systems for the development of ASF vaccine candidates.

## Materials and Methods

### Ethical approval

The study was approved by Local Ethics Committee of Kazan State Academy of Veterinary Medicine, Kazan, Russia (approval No. 04/08.22).

### Study period and location

The study was conducted from September 2022 to March 2023 in the Interdepartmental Laboratory of Immunology and Biotechnology, Kazan State Academy of Veterinary Medicine, Kazan, Russia.

### Genetic constructs

Nucleotide sequences of the genes of the epidemiologically significant isolate Georgia 2007/1 (GenBank ID NC_044959.2) *B646L* (p72), *CP530R* (pp60), *E183L* (p54), and *CP204L* (pp30) were used to construct codon-optimized genes that were pairwise cloned into the pAAV-MCS vector with a cytomegalovirus promoter, a linker for processing the 2A peptide in the intercistronic region, and a polyadenylation signal. Considering that the capacity of AAV is limited to 4.7 kb, the target genes in the bicistronic vectors were combined as follows: *E183L–CP204L* (1,235 bp), *B646L–E183L* (2,569 bp), and *CP530R–CP204L* (2,198 bp). The correctness of the sequences of the generated constructs was confirmed by sequencing.

### Oligonucleotide primers

Primers flanking the *B646L*, *CP530R*, *E183L*, and *CP204L* gene regions and primers flanking the AAV serotype 2 (AAV2) inverted terminal repeat (ITR) sequences were designed using the Vector NTI 9.0 software package (Life Technologies, USA), taking into account the absence of potential non-specific secondary structures (i.e., dimers and hairpins with high melting points). The specificity of the selected primer sequences was assessed using the NCBI BLAST online tool (http://www.ncbi.nlm.nih.gov/BLAST/). The sequences of the calculated oligonucleotide primers are presented in Table-1.

**Table-1:** Specific oligonucleotides flanking regions of the ASFV target genes and ITR AAV2.

Gene	Oligonucleotide	Sequence 5′ → 3′	Melting t,°C
*B646L*	Fp	GAAGGGAATGGATACTGAGGGAA	55.1
	Rp	CTCTCACAATATCCAAACAGCAGGTA	55.7
*CP530R*	Fp	AGACCTTAGAATCACTCATCCTTCCA	55.6
	Rp	AGATAGGGATTATTGACACTACACCAGTT	55.4
*E183L*	Fp	AGCAAGTGTAGGCAAGCCAGTC	55.2
	Rp	AGTGTTCTGAGTAGTGACTGTCGTGTAAG	55.7
*CP204L*	Fp	CAAGTGTAGGCAAGCCAGTC	55.1
	Rp	GCCATGACTAGTCTGTCCGT	55.6
*ITR*	Fp	GGAACCCCTAGTGATGGAGTT	54.0
	Rp	CGGCCTCAGTGAGCGA	56.0
	Probe	FAM-CACTCCCTCTCTGCGCGCTCG-BBQ	62.0

ASFV=African swine fever virus, ITR=Inverted terminal repeat, AAV2=Adeno-associated virus serotype 2

### Assembly of recombinant AAV2 (rAAV2)

AAV293 cells (ATCC^®^ CRL 3216™, USA), which are human embryonic kidney cells that have been optimized for the assembly of AAV virions, were cultured in Dulbecco’s modified eagle medium supplemented with 10% fetal bovine serum (HyClone, Australia), 100 IU/mL of penicillin and streptomycin (Gibco, USA), and 200 mM L-glutamine (Sigma-Aldrich, USA) in culture flasks (175 cm^2^) at 37°C in a 5% CO_2_ atmosphere to achieve a 70%–80% confluent monolayer at an initial seed concentration of 4 × 10^5^ cells/mL. Cotransfection of the monolayer with plasmids with the gene of interest, pAAV-RepCap, and pAAV-Helper was performed using the calcium phosphate method. Cells were incubated for 6 h, then the medium was changed, and the incubation was continued for up to 72 h. The transfection efficiency was assessed by fluorescence microscopy (Olympus, Japan) according to the fluorescence intensity of the far-red reporter protein TurboFP635 (Katushka 2S) (Evrogen, Russia). At the end of the incubation period, the transfected cells were removed mechanically and subjected to five cycles of freezing-thawing. The resulting virus-containing samples were concentrated by ultracentrifugation in a step density gradient of iodixanol.

### Determination of AAV2 titer

Samples of virus-containing material were treated with nuclease benzonase at a rate of 50 U/mL (Sigma-Aldrich), with an exposure of 30 min at 37°C to remove non-encapsidated DNA. Samples were maintained at 95°C for 5 min to inactivate benzonase. The number of collected virions, as well as confirmation of the presence of target genes in their composition, was determined by real-time polymerase chain reaction (PCR) on a C1000 amplifier with a CFX-96 optical unit (Bio-Rad, USA) according to the following amplification program: (I) DNA denaturation at 95°C for 3 min and (II) 40 cycles of 15 s at 95°C and 30 s at 57°C, with FAM fluorescence detection on each cycle at 57°C. The PCR results were recorded by electrophoretic separation in 1% agarose gel in TBE buffer at a voltage of 0.6 V/cm^2^ for 30 min followed by detection under ultra violet light after staining with ethidium bromide. The GeneRuler 1-kb Plus DNA ladder marker (Thermo Fisher Scientific, USA) was used with a range of fragments ranging from 75 to 20,000 bp in length as a standard for determining the lengths of the obtained amplicons. The presence of AAV2 virions was confirmed by electrophoresis in 12.5% polyacrylamide gel by the presence of the structural viral proteins VP1, VP2, and VP3.

### Evaluation of the functionality of the developed constructs

The functionality of the developed constructs was evaluated by the specific expression of the transgene (determination of the relative levels of messenger RNA [mRNA]) and by the presence of expression products in cell lysates (mature proteins p72, p30, p54, and pp62). Transduction of HEK293 and SPEV cell lines was performed at a multiplicity of infection (MOI) of 2000 genome copies per cell when the monolayer reached 70%–80% confluence. After the addition of pAAV2, the cells were incubated for 6 h, after which the virus-containing medium was removed, the monolayers were washed 3 times with phosphate buffer to remove non-adsorbed virions, and a maintenance medium was added. Total RNA was isolated from the cells using TRI reagent, and the upper colorless aqueous phase was removed after precipitation. After measuring the RNA concentration, a 1-μg matrix was used for complementary DNA synthesis. After reverse transcription, real-time PCR was performed using the above primers and the 10-fold serially diluted plasmids with the corresponding transgenes as references.

### Statistical analysis

The significance of the statistical difference between the mean values was determined by Student’s t-test using GraphPad Prism v.8.4 software (www.graphpad.com, GraphPad Software, CA, USA). Differences in values were considered significant at a significance level of p < 0.05. The data were presented as the mean ± standard error of the mean.

## Results and Discussion

### Production of rAAV2

Based on the results of the bioinformatics analysis (number of immunogenic epitopes, type and severity of the immune response, and its presence in target animals), the p54, p30, p72, and pp62 proteins showed the maximum potential, while p54, p30, and p72 were characterized mainly by B-cell type immune responses. In all proteins, except p72, there was no transmembrane domain, which indicated the extracellular localization of the molecules. The sequences of the *B646L*, *CP204L*, *E183L*, and *CP530R* genes, encoding the target proteins p72, p30, p54, and pp62, respectively, were codon-optimized *in silico*, with the most common codons of the recipient organism (pig) being used as the optimal codons. Outsourced genes (Evrogen) containing the transgene and its regulatory elements flanked by ITRs were pairwise cloned into the pAAV-MCS target vector. The *E183L* and *CP204L* genes were combined, since the p54 and p30 proteins are involved in the internalization of ASFV into the cell, and p72 and pp62 are the dominant structural viral proteins and, therefore, targets for serological diagnostics. The creation of such bicistronic constructs, in our opinion, under the condition of effective coexpression of transgenes, can reduce the potential vector load on the body during *in vivo* tests and generally optimize the production of AAV particles. In our AAV assembly protocol, it was found that in all samples, the titer of the recombinant virus according to ITR was at least 280 billion viral particles/mL. The presence of the corresponding target genes of ASFV was confirmed by PCR. In addition, the presence of rAAV2 in the samples was evaluated by protein electrophoresis in 12.5% polyacrylamide gel, and the major capsid protein pAAV2 VP3, with a molecular weight of 62 kDa, was visualized.

### Transduction of HEK293 cells by rAAV2

**Figure-1:**
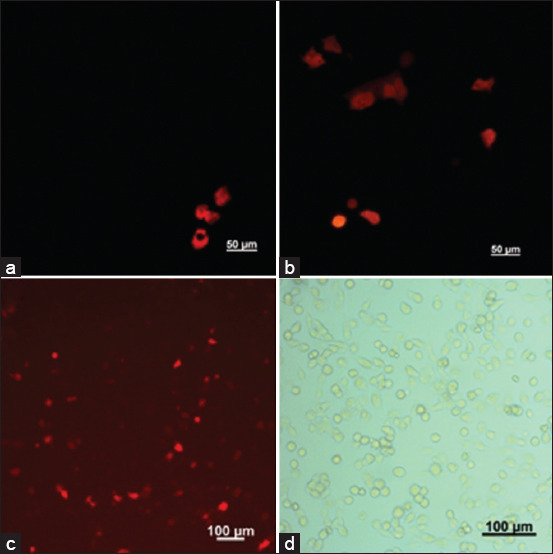
Fluorescence microscopy of the cells transduced with recombinant adeno-associated virus serotype 2-TurboFP635 (on the example of HEK293 cells): (a) 24 h post-transduction, (b) 48 h post-transduction, (c) 72 h post-transduction, and (d) light microscopy of a monolayer.

The next research stage was the implementation of cell transduction with the developed pAAV2 to further evaluate its functionality. Adeno-associated virus transduction requires binding to cell surface glycan receptors, which mediates the attachment of virions to the cell surface and triggers subsequent internalization. The cellular capture of AAV particles by endocytic vesicles is mediated by integrins and various transmembrane receptors, and internalization occurs through clathrin-mediated endocytosis. Human embryonic kidney cell lines (HEK293, ATCC^®^ CRL-1573™), which were used to evaluate the functionality of rAAV, were chosen for the transduction experiments. In addition, taking into account the nephrotropism of AAV serotype 2, we also included a cell line of porcine embryonic kidneys (SPEV, Collection of Cell Cultures of Vertebrate, Russia) in the experiment. Specific fluorescence was observed in all transduced cells after 24 h, indicating the expression of the far-red Katushka2S protein. After 48 and 72 h, the number of transduced cells increased significantly (Figure-1).

Based on the obtained results, it was confirmed that rAAV2 could effectively transduce both HEK293 and SPEV cell lines.

### Assessment of potential cytopathogenic effect of rAAV2

One of the main requirements for rAAV2 as a component of a candidate vaccine is its safety, that is, the absence of any cytopathogenic or cytotoxic effects on host cells. To optimize the transduction process and establish the optimal MOI, cells from the above lines were transduced with bicistronic rAAV2 constructs at MOIs ranging from 1000 to 500,000 genome-containing viral particles. After 72 h, the cells were removed mechanically and subjected to apoptosis flow cytometry. For cytofluorimetric detection, we used double staining with 3,3’-dihexyloxacarbocyanine iodide (DiOC_6_(3), Thermo Fisher Scientific), a membrane-tropic voltage-dependent dye, and propidium iodide (PI, Invitrogen, USA), a membrane-impermeable DNA stain. Staining ensured the division of cells into the following subpopulations: Viable cells; subpopulation Q4 (DiOC_6_^+^/PI^–^), cells in a state of late apoptosis; subpopulation Q1 (DiOC_6_^–^/PI^+^), cells in a state of early apoptosis; and subpopulation Q3 (DiOC_6_^–^/PI^–^). The cells mechanically destroyed during manipulation (subpopulation Q2) were excluded from the distribution. Flow cytometry revealed that a significant escalation in MOI (up to 100,000 genome copies per cell) did not induce apoptotic changes in the cells of any of the studied lines. After the incubation period, the total proportion of early, late apoptotic, and necrotic cells did not exceed 4.5%, which was comparable to the level in the negative control (intact cells).

In addition, no significant statistical differences were found between cell samples transduced with monocistronic and bicistronic constructs. These data indicate the absence of a cytopathogenic/cytotoxic effect in all developed rAAV2. It was also found that MOIs over 5000 did not significantly increase the level of transgene expression. In addition, to increase the level of transduction, protamine sulfate was added to the virus-containing material at a concentration of 10 μg/mL. It was found that its use increased the level of transgene expression by 12.7% ± 2.1% in HEK293 cells and by 8.5% ± 1.8% in SPEV cells.

### Evaluation of the efficiency of target gene expression in host cells

**Figure-2:**
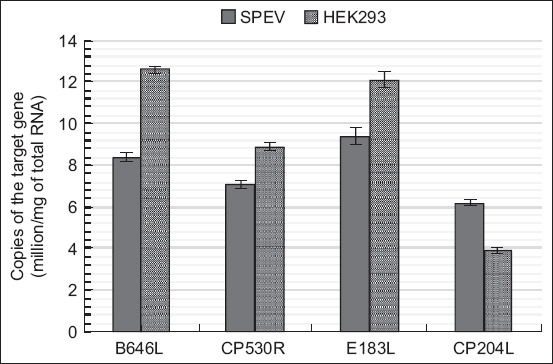
Messenger RNA expression levels during transduction of different cell lines with bicistronic constructs.

As a result of these experiments, it was found that the highest relative level of expression of specific mRNAs was achieved during transduction of SPEV cells and averaged from 7.1 to 12.6 million copies of the target gene/1 μg of total RNA. It was found that in the bicistronic constructs, the upstream cistron was expressed comparably to the same transgene in the monocistronic constructs, whereas the level of expression of the downstream cistron was slightly lower and varied from 3.5 to 3.9 million copies of the target gene/1 μg of total RNA (Figure-2). However, considering the presence of the corresponding mature proteins in the cell samples, the functionality of the downstream cistron was assessed as positive. In any case, when evaluating the properties of these rAAV2 *in vivo*, the immunization of pigs will be performed with both types of constructs.

## Conclusion

The prospect of obtaining economical and efficient vectors served as an incentive for the creation of polycistronic vectors in this study. Recombinant AAV serotype 2 was obtained using a new set of biotechnological tools based on the expression vector pAAV-MCS and key genes of ASFV. It was found that the developed constructs were able to effectively transduce both HEK293 and SPEV cell lines, which was confirmed by fluorescent expression control of the pAAV-Katushka2S. It was also noted that the proposed construct did not induce cytotoxic or cytopathogenic effects: according to the results of flow cytometry, the proportion of apoptotic cells during transduction did not exceed 4.5%. It was shown that the highest level of expression of specific mRNAs was achieved by transduction of SPEV cells and averaged 12.6 million copies of the target gene per 1 mg of total RNA. Meanwhile, the addition of protamine sulfate at a concentration of 10 μg/mL led to an 8.5% (±1.8%) increase in the expression level. Further, study should aim to test the developed genetic constructs *in vivo*. Special attention should be paid to the analysis of safety and possible side effects from the introduction of transgenic vectors and to the assessment of their impact on the immune status of pigs.

## Authors’ Contributions

RR: Designed and supervised the study and performed data collection. AG: Performed the experiments, data collection and analysis, and wrote the first manuscript. GF and EZ: Performed the experiments and investigated the study. ME and AR: Conceptualized and supervised the study, conceived the idea, and edited the manuscript. AH, LG, and DM: Collected and analyzed the data and drafted and revised the final manuscript. All authors have read, reviewed, and approved the final manuscript.

## References

[1] Ravilov R.K, Rizvanov A.A, Mingaleev D.N, Galeeva A.G, Zakirova E.Y, Shuralev E.A, Rutland C.S, Khammadov N.I, Efimova M.A (2022). Viral vector vaccines against ASF:Problems and prospectives. Front. Vet. Sci.

[2] Lacasta A, Ballester M, Monteagudo P.L, Rodríguez J.M, Salas M.L, Accensi F, Pina-Pedrero S, Bensaid A, Argilaguet J, López-Soria S, Hutet E, Le Potier M.F, Rodríguez F (2014). Expression library immunization can confer protection against lethal challenge with African swine fever virus. J. Virol.

[3] Lokhandwala S, Petrovan V, Popescu L, Sangewar N, Elijah C, Stoian A, Olcha M, Ennen L, Bray J, Bishop R.P, Waghela S.D, Sheahan M, Rowland R.R.R, Mwangi W (2019). Adenovirus-vectored African swine fever virus antigen cocktails are immunogenic but not protective against intranasal challenge with Georgia 2007/1 isolate. Vet. Microbiol.

[4] Mazur-Panasiuk N, Woźniakowski G, Niemczuk K (2019). The first complete genomic sequences of African swine fever virus isolated in Poland. Sci. Rep.

[5] Li D, Yang W, Li L, Li P, Ma Z, Zhang J, Qi X, Ren J, Ru Y, Niu Q, Liu Z, Liu X, Zheng H (2021). African swine fever virus MGF-505-7R negatively regulates cGAS-STING-mediated signaling pathway. J. Immunol.

[6] Arias M, Jurado C, Gallardo C, Fernández-Pinero J, Sánchez-Vizcaíno J (2018). Gaps in African swine fever:Analysis and priorities. Transbound. Emerg. Dis.

[7] Karger A, Pérez-Núñez D, Urquiza J, Hinojar P, Alonso C, Freitas F.B, Revilla Y, Le Potier M.F, Montoya M (2019). An update on African swine fever virology. Viruses.

[8] Cackett G, Matelska D, Sýkora M, Portugal R, Malecki M, Bähler J, Dixon L, Werner F (2020). The African swine fever virus transcriptome. J. Virol.

[9] Neilan J.G, Zsak L, Lu Z, Burrage T.G, Kutish G.F, Rock D.L (2004). Neutralizing antibodies to African swine fever virus proteins p30, p54, and p72 are not sufficient for antibody-mediated protection. Virology.

[10] O'Donnell V, Risatti G.R, Holinka L.G, Krug P.W, Carlson J, Velazquez-Salinas L, Azzinaro P.A, Gladue D.P, Borca M.V (2017). Simultaneous deletion of the 9GL and UK genes from the African swine fever virus Georgia 2007 isolate offers increased safety and protection against homologous challenge. J. Virol.

[11] Sunwoo S.Y, Pérez-Núñez D, Morozov I, Sánchez E.G, Gaudreault N.N, Trujillo J.D, Mur L, Nogal M, Madden D, Urbaniak K, Kim I.J, Ma W, Revilla Y, Richt J.A (2019). DNA-protein vaccination strategy does not protect from challenge with African swine fever virus Armenia 2007 strain. Vaccines (Basel).

[12] Netherton C.L, Goatley L.C, Reis A.L, Portugal R, Nash R.H, Morgan S.B, Gault L, Nieto R, Norlin V, Gallardo C, Ho C.S, Sánchez-Cordón P.J, Taylor G, Dixon L.K (2019). Identification and immunogenicity of African swine fever virus antigens. Front. Immunol.

[13] Jancovich J.K, Chapman D, Hansen D.T, Robida M.D, Loskutov A, Craciunescu F, Borovkov A, Kibler K, Goatley L, King K, Netherton C.L, Taylor G, Jacobs B, Sykes K, Dixon L.K (2018). Immunization of pigs by DNA prime and recombinant vaccinia virus boost to identify and rank African swine fever virus immunogenic and protective proteins. J. Virol.

[14] Goatley L.C, Reis A.L, Portugal R, Goldswain H, Shimmon G.L, Hargreaves Z, Ho C.K, Montoya M, Sánchez-Cordón P.J, Taylor G, Dixon L.K, Netherton C.L (2020). A pool of eight virally vectored African swine fever antigens protect pigs against fatal disease. Vaccines (Basel).

[15] Sang H, Miller G, Lokhandwala S, Sangewar N, Waghela S.D, Bishop R.P, Mwangi W (2020). Progress toward development of effective and safe African swine fever virus vaccines. Front. Vet. Sci.

[16] Dunbar C.E, High K.A, Joung J.K, Kohn D.B, Ozawa K, Sadelain M (2018). Gene therapy comes of age. Science.

[17] Doerfler P.A, Byrne B.J, Clément N (2014). Copackaging of multiple adeno-associated viral vectors in a single production step. Hum. Gene Ther. Methods.

[18] Shaimardanova A.A, Kitaeva K.V, Abdrakhmanova I.I, Chernov V.M, Rutland C.S, Rizvanov A.A, Chulpanova D.S, Solovyeva V.V (2019). Production and application of multicistronic constructs for various human disease therapies. Pharmaceutics.

